# Egg laying rather than host quality or host feeding experience drives habitat estimation in the parasitic wasp *Nasonia vitripennis*

**DOI:** 10.1002/ece3.5838

**Published:** 2019-11-22

**Authors:** Mareike Koppik, Andra Thiel, Thomas S. Hoffmeister

**Affiliations:** ^1^ Institute of Ecology University of Bremen Bremen Germany

**Keywords:** decision‐making, egg laying decision, egg load, information use

## Abstract

In variable environments, sampling information on habitat quality is essential for making adaptive foraging decisions. In insect parasitoids, females foraging for hosts have repeatedly been shown to employ behavioral strategies that are in line with predictions from optimal foraging models. Yet, which cues exactly are employed to sample information on habitat quality has rarely been investigated. Using the gregarious parasitoid *Nasonia vitripennis* (Walker; Hymenoptera: Pteromalidae), we provided females with different cues about hosts to elucidate, which of them would change a wasp's posterior behavior suggesting a change in information status. We employed posterior clutch size decisions on a host as proxy for a female's estimation of habitat quality. Taking into account changes in physiological state of the foraging parasitoid, we tested whether different host qualities encountered previously change the subsequent clutch size decision in females. Additionally, we investigated whether other kinds of positive experiences—such as ample time to investigate hosts, host feeding, or egg laying—would increase a wasp's estimated value of habitat quality. Contrary to our expectations, quality differences in previously encountered hosts did not affect clutch size decisions. However, we found that prior egg laying experience changes posterior egg allocation to a host, indicating a change in female information status. Host feeding and the time available for host inspection, though correlated with egg laying experience, did not seem to contribute to this change in information status.

## Graphical abstract

Habitat quality estimation enables female parasitoids foraging for hosts to make optimal foraging decisions. Here, we show that *Nasonia vitripennis* females use the experience of egg laying rather than mere host encounter or feeding on a host to update their habitat quality estimate. Furthermore, we find that, contrary to our expectations, females do not integrate information on host quality with other possible cues when estimating habitat quality.

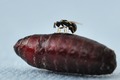

## 1 INTRODUCTION

To behave optimally in variable environments, foraging individuals have to estimate the quality of their habitat (Ydenberg, Brown, & Stephens, 2007). They may achieve this by relying on cues about the distribution and profitability of resources encountered in the past. Clutch size decisions in insects such as parasitoids can be treated as optimal foraging problems (Iwasa, Suzuki, & Matsuda, 1984; Mangel, 1987; Skinner, 1985). Similar to prey items, oviposition sites can be randomly distributed in the environment and may vary greatly in their profitability (Mangel, 1987); thus, ovipositing insects need plastic behavioral responses to adapt to their current habitat's quality. The close link between foraging success and lifetime fitness gain in parasitoid females (Godfray, 1994) has made them attractive model systems to investigate research questions on optimal foraging. We here use clutch size decisions of the pteromalid wasp *Nasonia vitripennis* to elucidate, which cues are used by females to estimate habitat quality.

Building on a landmark study on birds by Lack (1947), optimal clutch size per oviposition bout has been studied in a variety of insect species considering the fecundity of resulting offspring instead of their mere survival (Charnov & Skinner, 1984; Godfray, Partridge, & Harvey, 1991; Mangel, Rosenheim, & Adler, 1994). Therein, optimal clutch size is the number of eggs that maximizes the fitness gain of the ovipositing female per oviposition bout. However, even within this clutch size a female's fitness gain per egg decreases with increasing number of eggs added to a clutch, since the host represents a limited resource for the developing offspring. Depositing eggs on a new host instead, would thus increase a female's fitness gain per egg. Depending on which combination of factors ultimately limits a female's lifetime fitness (host availability, eggs or time), optimal clutch size maximizing lifetime fitness might therefore deviate from optimal clutch size maximizing fitness per oviposition bout/host. Here, we use the term optimal clutch size as the clutch size maximizing females' lifetime fitness gain, since females should be selected to optimize their overall fitness. Theoretical models maximizing lifetime fitness gain are based on models of optimal clutch size and have been expanded to match the purposes of insects foraging for oviposition sites, by including, for example, egg load and mortality into the models (Iwasa et al., 1984; Mangel, 1987, 1989; Mangel & Heimpel, 1998) using rate‐maximization as well as dynamic state‐variable approaches. One prediction of such models is that in parasitoids, maximizing lifetime fitness gain should lead to decreasing exploitation (smaller clutch sizes) of single hosts with increasing overall habitat quality. And such patterns have been found in empirical studies (Bezemer & Mills, 2003; Rosenheim & Rosen, 1991).

In female parasitoids, many studies on optimal foraging have been conducted by manipulating parameters indicating habitat quality that should trigger behavioral changes in foragers. Most of these studies have concentrated on patch time allocation as many parasitoids are deemed limited by the time they have available to search for hosts (Wajnberg, 2006). Thus, in these species time is a valuable resource that is under selection to be used optimally (rate‐maximization). In contrast, substantially fewer studies have been testing the effect of habitat parameters on clutch size decisions (but see Bezemer & Mills, 2003; Rosenheim & Rosen, 1991). Optimal clutch size decisions should equally be selected for in order to optimize lifetime reproductive success. Even fewer studies so far have explicitly dealt with testing, which of two or more alternative cues are employed when females track changes in habitat quality. For example, *Venturia canescens* (Hymenoptera: Ichneumonidae) either uses the energy expenditure during flight or the waiting time between patch visits as a cue for patch distance (Liu, Bernstein, & Thiel, 2009), depending on whether females come from thelytokous populations living in anthropogenic habitats or from arrhenotokous populations living under field conditions (Liu et al., 2009). Furthermore, habitat quality estimation in this species seems to be based on the number of eggs laid on previous patches, but not on the kairomone level therein (Froissart, Bernstein, Humblot, Amat, & Desouhant, 2012), even though under natural conditions both cues indicate the number of hosts being present.

Manipulations of habitat quality often go along with changes in the physiological state of an individual, impeding studies on the influence of information on decision‐making processes in insects (Rosenheim & Rosen, 1991); and only a few studies disentangled these two factors successfully. One way to achieve this has been used in an experiment on clutch size decisions in *Aphytis lingnanensis* Compere (Hymenoptera: Aphelinidae). The study revealed physiological state and experience (information) based decisions, by manipulating egg load through size and rearing temperature of experimental females (Rosenheim & Rosen, 1991). Another approach has been taken in studies on the effect of previous patch quality on habitat quality estimates in the parasitic wasp *Asobara tabida* (Hymenoptera: Braconidae), where habituation or egg load as forces potentially driving subsequent foraging decisions had been eliminated statistically (Thiel & Hoffmeister, 2006). A further study on *A. tabida* indicates that learning and the use of short‐term memory is involved in habitat quality estimation in this wasp (Louapre & Pierre, 2012), clearly suggesting an information based mechanism.

We here investigated, which cues may be employed during foraging to estimate habitat quality, using the gregarious parasitoid *N. vitripennis* (Hymenoptera: Pteromalidae) as a model system. We aimed to manipulate females' habitat quality by offering them a prior experience with hosts of different quality. Different habitat qualities can be achieved in many ways, by either manipulating number or quality of hosts, or both. As handling time of hosts is quite long for *N. vitripennis*, we chose to manipulate the quality of a single host rather than the number of hosts within our experiment. We aimed to test, which cue (i.e., time available to investigate hosts, experience with host feeding, or experience with laying eggs) is needed to trigger changes in posterior egg allocation decisions. These clutch size decisions were taken as a proxy for habitat quality estimation, since optimality models suggest that increasing habitat quality should lead to decreasing exploitation of hosts. We statistically controlled for initial clutch size to avoid confounding effects of this influential physiological state variable. Our main hypothesis was that host quality would be the main cue used by the females to estimate habitat quality and that experience with poorer quality hosts would lead to lower estimates for habitat quality. More complete information about hosts indicated by the time available for host inspection, host feeding, or egg laying, respectively, might be involved in changes in habitat quality estimates in females. For instance, host quality might change habitat quality estimates when females spend 2 hr inspecting the host, but not when they were only allowed to briefly drill into that host. Alternatively, habitat quality estimate changes, due to differences in quality of previously encountered hosts, might only occur when females fed on those hosts or laid eggs.

## 2 MATERIALS AND METHODS

### 2.1 Study organisms

**Figure 1:**
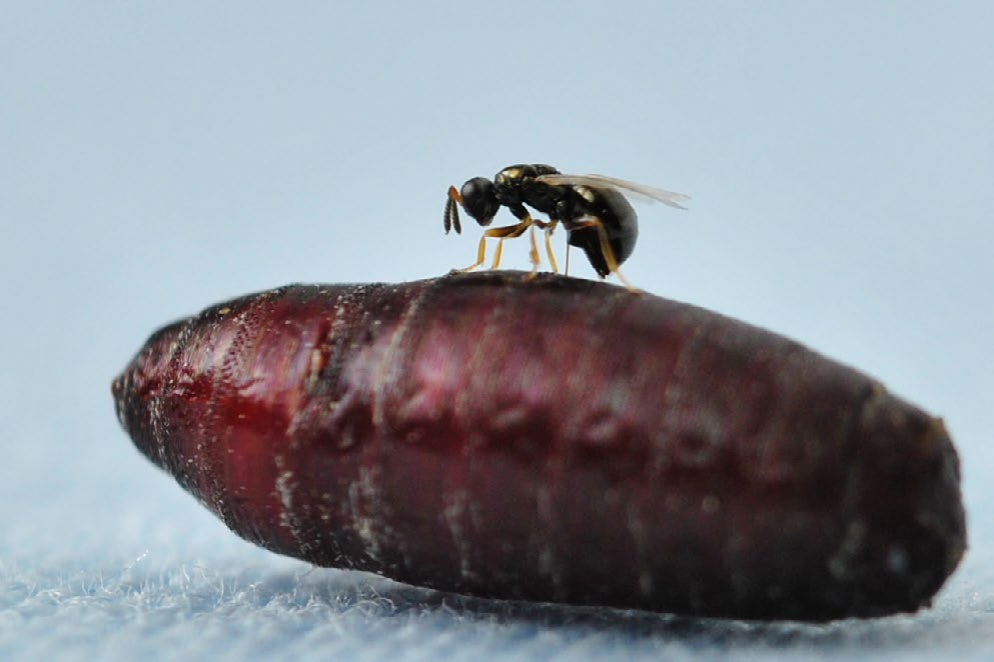
*Nasonia vitripennis* female drilling into a host pupa (*Calliphora vomitoria*)

We used *N. vitripennis* and its host *Calliphora vomitoria* L. (Diptera: Calliphoridae) as a study system (Figure 1). *Nasonia vitripennis* is a gregarious parasitoid, 1.0–3.5 mm in size, attacking pupae of a wide range of cyclorraphous Diptera (Whiting, 1967). For the experiments, we used the *N. vitripennis* laboratory strain HVRx, which was established from five different lines collected in 2001 in the Hoge Veluwe area (Netherlands) and cultured to maintain high genetic variability (van de Zande et al., 2014). Wasps were kept in an incubator at 25°C and 60% r.h. under a L16:D8 regime and reared on fly pupae of *C. vomitoria*. Hosts were obtained as maggots from a local pet shop and kept at 25°C in saw dust until 2 days after pupation. Afterward, they were transferred to 4°C until use.

Female wasps were sorted as pupae and kept individually in gelatine capsules (0.37 cm^3^) until hatching. Freshly emerged females were kept singly in polystyrene vials (27 × 60 mm) and fed 10% (vol/wt) sugar solution. On the first day, every female was kept with a male to allow mating. On the second to fourth day, females had access to one fresh standardized host (67.5–72.5 mg) each day; on the fourth day, however, the host was removed after 4 hr and females were deprived of hosts (for 18–20 hr) until the start of the experiment on the next day. This procedure allows females to mature and accumulate eggs. *Nasonia vitripennis* is a concurrent host feeder (Rivero & West, 2005) and egg maturation and resorption is strongly dependent on host feeding (Edwards, 1954; Richard & Casas, 2012). Female wasps were used for experiments 5 days after hatching.

For the experiments, hosts were weighed on the day of pupation. We created three different host qualities for prior experience of the females. We here define host quality as the potential fitness an ovipositing female can gain from a given host. Host qualities were chosen to maximize differences between host qualities based on results from a previous study, which demonstrated that different offspring numbers eclose from hosts varying in parameters like size, age, and parasitization status and that this is a result of adaptive female decision‐making rather than offspring mortality (Koppik, Thiel, & Hoffmeister, 2014). Good quality hosts were obtained by using large hosts (82.5–87.5 mg) kept at 25°C until 2 days after pupation and stored at 4°C until use. Medium‐quality hosts were obtained by using medium sized hosts (67.5–72.5 mg) kept at 25°C until 1 day after pupation and parasitized by a female of a red‐eyed mutant strain for 4 hr 1 day before the experiment. Low quality hosts were obtained the same way, only that hosts were preparasitized 4 days before the experiment. Previous studies have shown that females avoid laying eggs on 4 days previously parasitized hosts and lay fewer eggs on 1 day previously parasitized hosts compared to unparasitized hosts, which therefore represent low‐ and medium‐quality hosts, respectively (King & Rafai, 1970; Koppik et al., 2014; Shuker, Pen, Duncan, Reece, & West, 2005; Werren, 1984). While eggs laid on 1 day previously parasitized hosts face heightened competition, the older and larger larvae present in 4 days previously parasitized hosts would most likely always fully outcompete all eggs laid at this point.

### 2.2 Experimental assay

**Figure 2:**
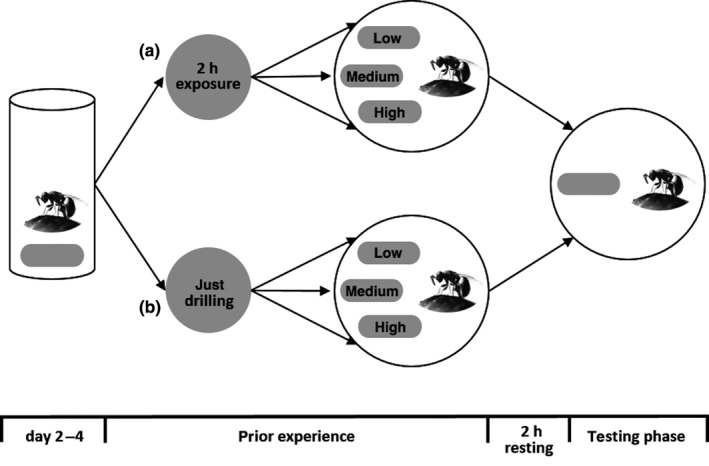
Schema of the experimental setup. Experimental females differed in their prior experience. One half of the females was exposed to a host (*Calliphora vomitoria*) of low medium or high quality for 2 hr (a). The other half of the females was only allowed to drill into a host (*C. vomitoria*), again, of low, medium, or high quality (b). After a 2‐hr resting period, clutch size decisions of all females were recorded on a second host which was of the same quality for all females (*C. vomitoria* pupa weighing 52.5–57.5 mg)

Experiments were conducted in a climatic chamber at a constant temperature of 25 ± 1°C. Experiments consisted of a prior experience and a testing phase (Figure 2). In both phases, females received a host in a closed petri dish (Ø 5 cm). The two phases were separated by a 2‐hr resting period during which females were kept singly in polystyrene vials (27 × 60 mm) and fed 10% (vol/wt) sugar solution.

During the prior experience, there were two different experience groups. In the first group (“only drilling”), females were only allowed to drill into a host and were afterward gently brushed off the host. Thus, we presume females could sense the quality of the host while not being able to host feed or lay eggs. In the second group (“2‐hr exposure”), females were allowed to spend 2 hr with the host, including the possibility for egg laying and host feeding. Within each experience group, females were divided into three host quality groups either receiving a high, medium, or low quality host during prior experience. During the prior experience phase, females were constantly observed and key behaviors, such as host feeding, were recorded using the software The Observer XT (Noldus).

During the testing phase, females from all six groups were tested in the same way. Each female received one host (52.5–57.5 mg) and was constantly observed until she decided to abandon the host (5 min off the host) or until 10 p.m. (end of light phase in the rearing incubator). Afterward, females were transferred to ice (to stop egg maturation) and at the end of the day frozen to determine the number of remaining mature eggs inside their ovaries. Egg load was defined as the number of eggs that were available to the female during the experimental phase: remaining mature eggs plus eggs laid on the testing host.

All hosts of the prior experience and testing phase were kept at 25°C for 15 days to count hatching offspring. Afterward, all hosts were carefully opened to also check for dead, diapausing, or not fully developed offspring, which were added to the total offspring count. In general, offspring mortality is very low in *N. vitripennis* (Koppik et al., 2014). Hosts from the testing phase contained dead, diapausing or not fully developed offspring only in 10 out of 118 cases. Incidents of nonemerged offspring decreased with increasing clutch size (GLM, binomial distribution, *N* = 118: *df* = 1, *χ* ^2^ = 8.19, *p* = .004), and we thus suspect some kind of Allee effect (Stephens, Sutherland, & Freckleton, 1999) rather than mortality due to lack of resources. Trials with hosts (from the prior experience or testing phase) that turned out to be unsuitable for parasitization were excluded from the analysis. Unsuitable hosts were defined as hosts, which were completely hollow, rotten, or dried up after 15 days without giving rise to any parasitoid offspring. We suspect that those were infected with bacteria that killed/consumed the host and possibly eggs/feeding larvae, thus we cannot be sure of females' clutch size decisions for these hosts. For each experience, host quality combination 18–21 replicates entered the analysis.

### 2.3 Statistical analysis

Statistics were performed in R 3.5.1 (R Core Team, 2018) using Generalized Linear Models (Fox & Weisberg, 2011); correlation coefficients have been calculated with package *psych* (Revelle, 2018). The respective error distributions used in the GLMs are provided with the test results. *p*‐values were obtained by comparing nested models using likelihood ratio tests, and nonsignificant terms were eliminated stepwise to arrive at the minimal adequate model. For post hoc pairwise comparisons with Bonferroni corrected *p*‐values, we employed package *multcomp* (Hothorn, Bretz, & Westfall, 2008). Graphs were made using the software R 3.5.1 (R Core Team, 2018) and package *gplots* (Warnes et al., 2016).

To test which factor influenced female estimation of their habitat quality, we used clutch sizes in the testing phase as a proxy for habitat quality estimates. Higher clutch sizes indicate females estimating their habitat to be of lower quality. Females were given different experiences on the first host (only drilling into/2‐hr exposure to either a low, medium, or high quality host), which led to females possibly differing in three states (time with host: 2 hr or drilling, previous egg laying: whether or not they had laid eggs on the first host, and previous host feeding: whether or not they had fed on the first host). However, these three states were highly correlated indicated by the correlation coefficients phi (previous egg laying and previous host feeding: 0.63, previous egg laying and time with host: −0.57, previous host feeding and time with host: −0.81). To test which of the three states (each a two‐level qualitative variable) best explained variation in female habitat quality estimation, we compared models using Akaike information criterion (AIC). This allowed us to directly compare models using either one of the three states as a predictor, since models do not have to be nested when using AIC (Zuur, Hilbe, & Ieno, 2013). For each of the three possible states, we constructed models including previous host quality (three‐level qualitative variable) as an interaction or additive term. Since females with different experiences should have different egg loads, egg load (quantitative variable) was always included in the models to control for effects derived from a female's egg state. Additionally, some trials (11 out of 118) were terminated by the experimenter at 10 p.m.; therefore, an additional variable “censored” (a two‐level qualitative variable indicating whether or not the trial was terminated by the experimenter) was introduced to correct for any influence of artificial termination of the experiment.

## 3 RESULTS

### 3.1 Influence of current host quality on clutch size during prior experience

**Figure 3:**
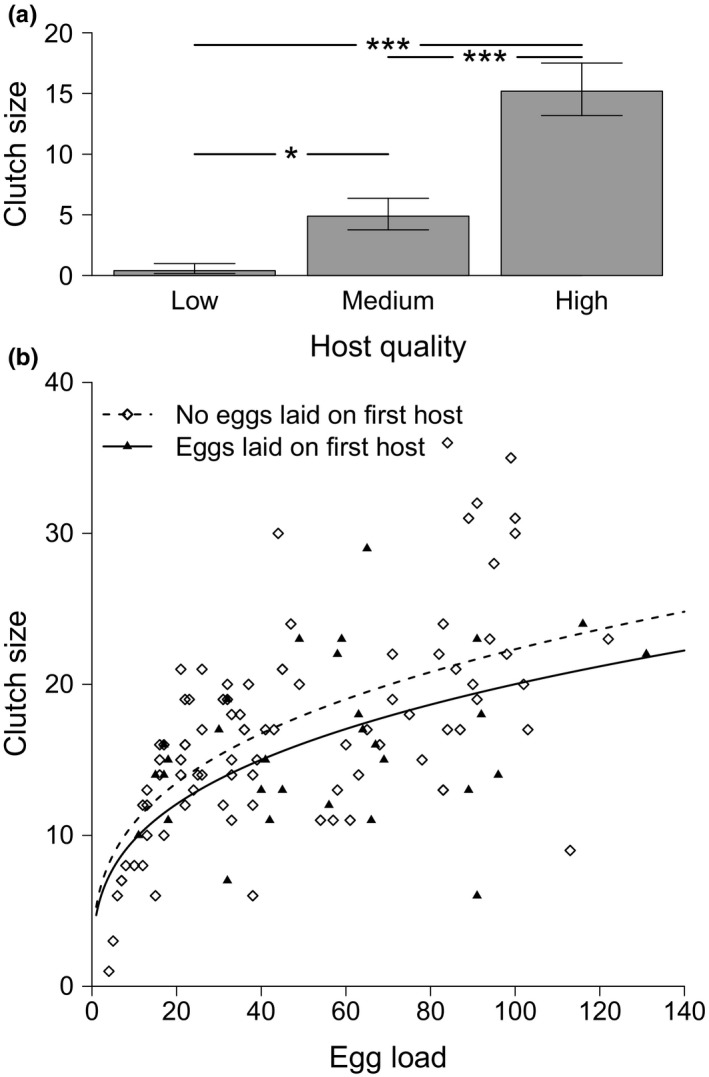
Female clutch size decisions. (a) During the 2 hr of prior experience, females laid significantly more eggs on high‐quality hosts than on lower quality hosts (*N* = 60, *p* < .001). Estimates (±*SE*) are derived from the statistical model. Symbols represent Bonferroni corrected outcomes of post hoc pairwise comparisons: *p < .05, ***p < .001. (b) Clutch size decisions of females during the testing phase as a function of their egg load. Females that had laid eggs on the previous host (filled triangles, solid line) produced smaller clutches on the current host compared to females that had not laid any eggs on the previous host (open diamonds, dashed line), raw data and regression lines derived from the statistical model

By allowing females to produce egg clutches during the 2‐hr exposure on hosts in the prior experience phase, we can test if females were able to sense the quality of the offered host and would respond with clutch size variation. We found females to lay significantly larger clutches on hosts of higher quality compared to lower quality hosts (Figure 3a). Overall, clutch size was significantly influenced by the quality of the current host (GLM with Poisson error distribution corrected for overdispersion, *N* = 60, *F*_2,57_ = 28.53, *p* < .001, deviance based $R_{D}^{2}$ = 0.50).

### 3.2 Factors influencing female habitat quality estimate

Surprisingly, the quality of the previous host did not influence female clutch size decisions (Table 1). Yet, females laid larger clutches of eggs on the testing host with increasing egg load and when having laid no eggs during prior experience (Figure 3b). Of the three possible female state variables, “previous egg laying” explained clutch size variation on the testing host best (Table 1). Termination of the experiment (censored) resulted in slightly smaller clutches.

**Table 1:** AIC values for different models of clutch size decisions on the second host (GLM, Poisson error distribution, *N* = 118)

Variable	*df*	AIC
Time with host × previous host quality + ln(egg load) + censored	8	739.68
Time with host + previous host quality + ln(egg load) + censored	6	737.08
Time with host + ln(egg load) + censored	4	733.86
Previous host feeding × previous host quality + ln(egg load) + censored	8	740.41
Previous host feeding + previous host quality + ln(egg load) + censored	6	738.60
Previous host feeding + ln(egg load) + censored	4	735.39
Previous egg laying × previous host quality + ln(egg load) + censored	8	735.88
Previous egg laying + previous host quality + ln(egg load) + censored	6	735.61
Previous egg laying + ln(egg load) + censored	**4**	**732.19**
Previous host quality + ln(egg load) + censored	5	737.76
ln(egg load) + censored	3	734.54

Note

Deviance based $R_{D}^{2}$ = 0.38 of the best model: clutch size ~ previous egg laying + ln(egg load) + censored.

Values in bold indicate the model best explaining variance in clutch size decisions on the second host (lowest AIC).

Within the group of females that had laid eggs during the prior experience, we found no significant effect of number of eggs laid on this host (which might be used as a fine‐scaled measurement of host quality (Froissart et al., 2012)) on clutch size on the testing host, when correcting for female egg load (GLM, Poisson distribution, *N* = 30: *df* = 1, *χ* ^2^ = 0.57, *p* = .450). This further supports the conclusion that *N. vitripennis* does not use previous host quality to estimate habitat quality.

## 4 DISCUSSION

There is ample evidence that female parasitoids use prior experiences to update their habitat quality estimate and adjust their foraging behavior (e.g., Bezemer & Mills, 2003; Liu et al., 2009; Rosenheim & Rosen, 1991; Thiel & Hoffmeister, 2004). Here, we have been interested to elucidate which cues may trigger an update in habitat quality estimates using the gregarious parasitoid *N. vitripennis*. As possible cues, we analyzed the time available for host inspection, host feeding experience, and egg laying experience on a previously encountered host of varying quality. As proxy for habitat quality estimation, we used the clutch size decisions on the testing host offered.

Females did not alter their clutch size decisions on the subsequent testing host in response to previous host quality (Table 1). This indicates that changes in host quality are not used by *N. vitripennis* females to change their estimate of habitat quality, which is in contrast to many other species studied (e.g., Bezemer & Mills, 2003; Louapre, Baaren, Pierre, & Alphen, 2011; Rosenheim & Rosen, 1991; Wajnberg, 2006). However, a similar phenomenon has been shown for the parasitic wasp *Lysiphlebus testaceipes*, where the first patch encounter seems to set the habitat quality estimate of a female rather than a constant updating of information during foraging (Tentelier, Lacroix, & Fauvergue, 2009). Low‐ and medium‐quality hosts in our study have been obtained by preparasitization of hosts, an attribute that changes subsequent foraging behavior in some species (*Leptopilina heterotoma* and *Monoctonus paulensis*, Roitberg et al., 1992; Michaud & Mackauer, 1995) but not in others (*A. tabida*, Thiel & Hoffmeister, 2006). Thus, whether information on host parasitization status is used by parasitoid females to estimate habitat quality appears to vary across species, and our results show that *N. vitripennis* does not use this information.

In contrast to *V. canescens*, where females adjust habitat quality estimation based on the number of eggs laid on previous patches (Froissart et al., 2012), we did not find such a mechanism in *N. vitripennis*. A female's clutch size decision was not influenced by the number of eggs laid on the previous host, but just by the fact whether or not she had laid eggs. As a generalist, *N. vitripennis* parasitizes a broad range of dipteran hosts that may be found on carcasses or birds' nests (Abraham, 1985; Voss, Spafford, & Dadour, 2009). These communities of flies can be mixed (Daoust, Savage, Whitworth, Belisle, & Brodeur, 2012) such that quality of hosts varies greatly even within one host patch (Peters, 2010). Accordingly, the quality of one host encountered might not have a high predictive value for the quality of other hosts around and might therefore not be used by females to estimate habitat quality.

Even though previous host quality is not used in habitat quality estimation, previous egg laying significantly changed a female's current clutch size decision. Since we corrected for current egg load in our analysis, these changes are most likely information driven. Females that had laid eggs into the previous host reduced their clutch size on the current host, indicating that they estimated their habitat to be of better quality than females that lacked this previous egg laying experience. Though egg laying was correlated with time spent with the previous host as well as with host feeding, the latter two did not explain the variation in clutch size on the second host as well as egg laying experience. Consequently, an egg laying event rather than mere time spent with the host or a host feeding event seems to have led to different estimates of habitat quality. As we did not include a group without any host encounter during the pre‐experience phase, we cannot exclude that females in all groups increased their habitat quality estimate compared to females that would have had no host encounter at all. However, if it were that habitat quality would merely be estimated based on host encounter rates, we would have expected to see no difference at all between females in our experiment, since all females have had the same number and frequency of host encounters. Yet, females seem to have valued the different types of experiences differently. In contrast to a mere host encounter or host feeding event, egg laying might be a more reliable indicator for the females that they have encountered a host suitable for oviposition. This would be a relatively simple cognitive solution to update habitat quality estimates. Rather than integrating information on number and quality of hosts encountered, females would simply increase their estimate of habitat quality with each egg laying event. Egg laying induces various changes in the transcriptome of *N. vitripennis* females, and at least some of these changes have been proposed to relate to neuronal processes (Pannebakker, Trivedi, Blaxter, Watt, & Shuker, 2013). The involvement of neuronal processes in egg laying indicates information processing that may well be also related to updating habitat quality estimates by females. Here, we cannot distinguish whether absolute number of hosts or the host encounter rate (integrating time in between egg laying events) is used by *N. vitripennis* females as a measure of habitat quality. Females of the parasitoid *Trichogramma euproctidis* have recently been shown to perceive time and use it in their decision‐making processes (Parent, Brodeur, & Boivin, 2016). *Nasonia vitripennis* females might thus integrate time between egg laying events or use time since last oviposition as a measurement of habitat quality (whereby an egg laying event triggers “starting the clock”).

To conclude, we showed that *N. vitripennis* females used an oviposition event on a host rather than the number of eggs laid or the experienced host quality to determine clutch sizes laid on a subsequent host. Thus, laying eggs on hosts seems to serve as an updating mechanism for habitat quality estimation. Clearly, the number of hosts that females oviposited onto indicates the availability of suitable hosts in a habitat and may provide wasps with a more precise estimate of habitat quality than host encounter rate or host qualities encountered. As has been shown in other species, current egg load and thus physiological state of a female interact with habitat quality estimation and must not be ignored when analyzing clutch size decisions in foraging parasitoids.

## CONFLICT OF INTEREST

None declared.

## AUTHOR CONTRIBUTIONS

MK, AT and TSH conceived the study and designed the experiments, MK collected and analyzed the data, MK, AT and TSH wrote the manuscript.

## Data Availability

The data sets used for this study are available at Dryad repository (https://doi.org/10.5061/dryad.qnk98sfbk).
